# Age, experience, social goals, and engagement with research scientists may promote innovation in ecological restoration

**DOI:** 10.1371/journal.pone.0274153

**Published:** 2023-04-25

**Authors:** Jakki J. Mohr, Tina M. Cummins, Theresa M. Floyd, Elizabeth Covelli Metcalf, Ragan M. Callaway, Cara R. Nelson

**Affiliations:** 1 College of Business and Institute on Ecosystems, University of Montana, Missoula, Montana, United States of America; 2 W.A. Franke College of Forestry and Conservation, University of Montana, Missoula, Montana, United States of America; 3 College of Business, University of Montana, Missoula, Montana, United States of America; 4 Division of Biological Sciences and Institute on Ecosystems, University of Montana, Missoula, Montana, United States of America; 5 Department of Ecosystem and Conservation Sciences, W.A. Franke College of Forestry and Conservation, University of Montana, Missoula, Montana, United States of America; State University of New York, REPUBLIC OF KOREA

## Abstract

Innovation in ecological restoration is necessary to achieve the ambitious targets established in United Nations conventions and other global restoration initiatives. Innovation is also crucial for navigating uncertainties in repairing and restoring ecosystems, and thus practitioners often develop innovations at project design and implementation stages. However, innovation in ecological restoration can be hindered by many factors (e.g., time and budget constraints, and project complexity). Theory and research on innovation has been formally applied in many fields, yet explicit study of innovation in ecological restoration remains nascent. To assess the use of innovation in restoration projects, including its drivers and inhibitors, we conducted a social survey of restoration practitioners in the United States. Specifically, we assessed relationships between project-based innovation and traits of the *individual practitioner* (including, for example, age, gender, experience); *company* (including, for example, company size and company’s inclusion of social goals); *project* (including, for example, complexity and uncertainty); and *project outcomes* (such as completing the project on time/on budget and personal satisfaction with the work). We found positive relationships between project-based innovation and practitioner traits (age, gender, experience, engagement with research scientists), one company trait (company’s inclusion of social goals in their portfolio), and project traits (project complexity and length). In contrast, two practitioner traits, risk aversion and the use of industry-specific information, were negatively related to project-based innovation. Satisfaction with project outcomes was positively correlated with project-based innovation. Collectively, the results provide insights into the drivers and inhibitors of innovation in restoration and suggest opportunities for research and application.

## Introduction

Innovation can offer important opportunities to achieve ambitious restoration objectives; however, new restoration approaches may be inherently risky. In order to provide insights for both research and practice, our research investigates practitioner perspectives to assess empirically the facilitators and barriers of innovation in restoration projects.

Innovation refers to a new idea, device, or method [1, 2] that may arise from novel recombination of existing ideas or the application of better solutions or unique approaches to solve existing problems [3]. Innovations can arise from many sources, including lessons learned from failed experiments, ideas from mavericks or “rogue” thinkers who inherently question everything, insights gleaned from adjacent endeavors, formal scientific research, and serendipity—the random “bolts of inspiration/insight” associated with genius inventors [4]. Similarly, innovations in ecological restoration can come from varied sources. For example, despite being controversial [5], challenging dogma and long-held assumptions about the way ecological restoration is conducted can stimulate new thinking [6]. Questions such as “should restoration always use native species?” [7] and “are local provenances always best?” [8] challenge assumptions in ways that can lead to innovation. Innovations in ecological restoration might also include the use of new technologies, such as drones or remote sensing that collect data more efficiently than labor-intensive techniques [9–12] and machine learning algorithms that improve decision making [13–15]. Innovations can arise from the use of new methods, such as novel approaches to growing seed stock [16] or leveraging genomics to address restoration concerns [17, 18]. Moreover, others advocate for bringing an entrepreneurial mindset to ecological restoration, such as regarding failure as a trigger for innovation [19; see also 5]. Indeed, “businesses [engaged in ecological restoration] are very well suited to fostering innovation in restoration,” and “the core values of many private firms can be aligned with innovation to support opportunistic tinkering” [20].

Innovation is crucial for both reversing high rates of environmental degradation that lead to the loss of biodiversity [21] and for achieving ambitious global targets for ecosystem restoration and repair (see the UN Decade on Ecosystem Restoration Report [22]). For instance, to meet commitments to restore millions of hectares of forests, innovation is identified as an urgent need for landscape-level planning and prioritization, seed sourcing and propagation, and monitoring success [23]. By bringing novel techniques and new approaches, innovation plays an important role in achieving goals for ecological restoration [6]. However, the factors that drive innovation in restoration are not well understood.

Despite its importance, innovation in restoration faces barriers and constraints. Resources for innovation are often absent or limited in ecological restoration compared to other industries such as agriculture, medicine, or business [6, 19]. Indeed, businesses must “navigate the many trade-offs and complexities in restoration projects” [24] to achieve ecological objectives while earning sufficient profit. Interestingly, despite its status as a dominant natural resource management activity, with over US $1 trillion spent annually in the global “restoration economy” [25], research in restoration ecology has rarely addressed the perspective of the businesses engaged in the practice of restoration (see [24] for an exception). Yet, due to the inherent uncertainty of and possible failure from trying novel techniques, ecological practitioners can be reluctant to try new things. One practitioner summed it up:

“…it is hard to do innovative things and still recognize the fact that down the road you’d be looked at as, was your project successful or not. In the end, that’s what people are interested in. They don’t really care if you use some innovative technology to get there or not” [24].

Thus, restoration practitioners who restore degraded ecosystems face a paradox in innovation: on one hand, innovation is imperative to meet the global challenges and mandates for ecological restoration; on the other hand, innovation faces barriers and constraints. The purpose of our research was to understand the correlates (e.g., facilitators and barriers) of innovation in restoration projects. To our knowledge, this is the first study to examine this issue empirically in a restoration context.

### Hypotheses

**Fig 1:**
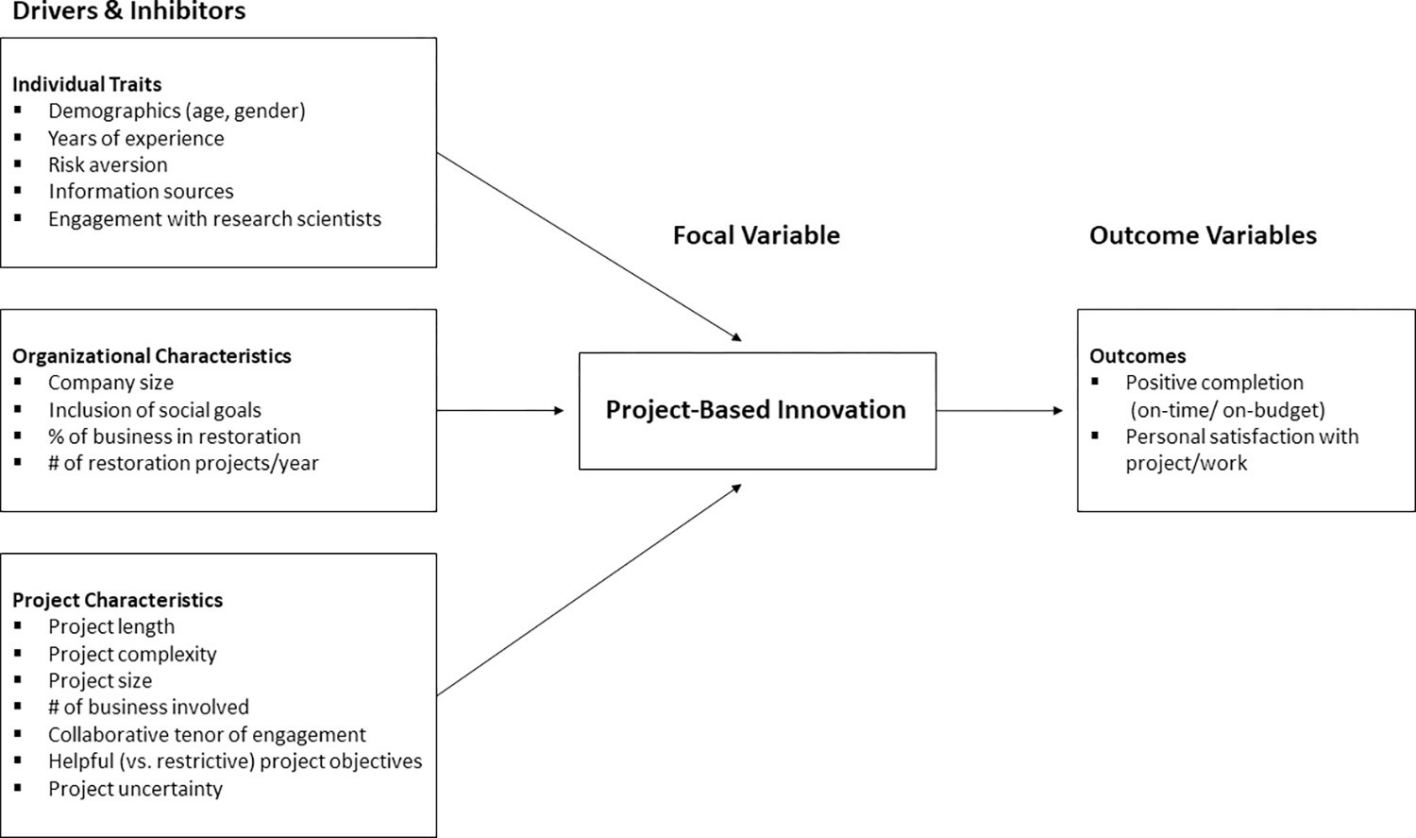
A model of innovation in ecological restoration.

To develop hypotheses, we examine related fields for insights. Business management and marketing have a long history of studying antecedents to innovation [26, 27]. Indeed, Rogers’ seminal work on the diffusion of innovations focused on the area of agricultural innovations [28]. More recently, the broad field of environmental management has explored issues related to the uptake and adoption of new practices (e.g., [29–31]). Based on this prior research, we grouped potential drivers and inhibitors into three categories (Fig 1), specific to the context of restoration: individual-level, company-level factors, and project-level traits. Our research explores two overarching research questions: (1) How do (a) individual traits and personal characteristics, (b) company/organizational traits and characteristics, and (c) project traits and characteristics relate to respondents’ perceptions of the degree of innovation used in their projects? In addition, we explore project outcomes relate to innovation: (2) How does project-based innovation relate to project outcomes, such as completing the project on-time/on-budget, as well as the respondents’ personal satisfaction with the work? Here, we review prior research to develop our hypotheses.

#### Individual traits

Individual traits such as age, years of experience, gender, as well as risk tolerance/aversion [29, 32], are related to adoption of innovation; we explore whether and how these factors are related to innovation in the restoration context. Past research in the organizational literature has yielded mixed results on the relationships between age/experience and innovation behavior. Some research finds that older, more experienced people are more confident in their expertise and, thus, are more comfortable trying new methods [33–35]. However, other research finds that younger, less experienced people bring up-to-date knowledge about innovative practices from their more recent schooling [36, 37]. Hence, although we hypothesize a relationship between age and years of experience and use of innovation, we do not hypothesize the direction of the relationship. Regarding gender, there is a clearer pattern in past research [26], which finds that men are more likely to engage in innovation than women [38]. Thus, we hypothesize that male restoration practitioners will be more likely to use innovative methods than female practitioners. Risk aversion refers to an individual’s preference for a sure outcome over a decision that has an uncertain outcome. Past research clearly indicates that people who prefer to avoid uncertain outcomes tend to be less innovative (e.g., [29, 31, 32]). In a qualitative study of the challenges businesses face in restoration [24], restoration practitioners stated that risk avoidance was one reason for not engaging in innovation. Thus, we hypothesize that risk aversion is negatively related to innovation in restoration projects.

In addition to these individual traits, the adoption of innovations is related to information sources that an individual uses [31, 39]. Another variable related to innovation is the extent to which practitioners engage with research scientists [40]. Because the science/practice nexus is also critical in ecological restoration [41, 42], we explore both the extent to which a practitioner engaged with research scientists as well as the practitioner’s perceptions about the degree to which such engagement was helpful. Given that restoration is a unique context, we do not offer specific hypotheses regarding these factors.

#### Company/organizational characteristics

The relationship between company/organizational characteristics and innovation is also well-established in the literature [27, 31], with company size being particularly well-studied. Although the business literature suggests that smaller companies tend to be more innovative and face fewer restrictions in using new ideas/techniques [43], the agricultural conservation literature suggest that larger operations are more likely to be innovative [31]. Again, given the unique context, we do not state a directional hypothesis regarding the relationship between company size and innovation in the restoration context.

Another organizational characteristic related to innovation is the degree to which the needs of users and other stakeholders are explicitly considered [44]. This orientation may be reflected in the degree to which restoration projects focus on social goals (in addition to ecological goals) (e.g., community engagement, recreation goals, economic livelihood [45, 46]). In this case, we hypothesize that explicit consideration of social goals will be positively associated with innovations.

Another organizational characteristic that can affect innovation is the ability of the organization to leverage its past experience for innovation [47]. We hypothesize that restoration businesses that have a greater percentage of their work in restoration and conduct more restoration projects are likely to be more innovative, because of their enhanced skills and competencies.

#### Project-level characteristics

Project characteristics also are related to innovation. Here, we examine project length, project size, project objectives, project complexity, and uncertainty surrounding the project. With respect to project length and project size, longer projects and larger projects may offer greater opportunities to try innovative practices. Hence, we hypothesize them to be positively related to innovation.

Research in business organizations indicates that project objectives may affect innovation. Qualitative research on the business of restoration [24] found that restoration practitioners may perceive project objectives to be encouraging/helpful (versus restrictive), with more restrictive objectives associated with relying on tried-and-true techniques because they are perceived as more likely to “get the job done.” Thus, in contrast to restrictive objectives, we hypothesize that helpful project objectives will be positively related to innovation.

Project complexity as well as uncertainty surrounding a project can also affect innovation. In business organizations, project complexity is negatively correlated with innovation [48]. Given the uncertain context in which restoration occurs, we also examined whether and how project uncertainty might affect the use of innovation in restoration projects.

Because project partners can be a source of new ideas [49], other project-related variables included number of other businesses involved, and the collaborative tenor of engagement with these other businesses.

#### Project outcomes

Businesses adopt innovations to achieve improved outcomes not attainable using existing methods, tools, and approaches. Because ecological restoration outcomes may take years to unfold, we focused on the degree to which a particular project was completed on time and on budget. As noted previously, because innovations can be risky, it is unclear whether they might result in project delays and/or cost over-runs. Moreover, we were curious about the relationship between innovation and the individual practitioner’s satisfaction with the work on the project. We view both relationships (between innovation and (a) completing projects on time/on budget and (b) the individual’s personal satisfaction) as exploratory, and do not offer directional hypotheses.

## Methods, sampling frame, and data collection

We designed and administered a questionnaire sent to restoration practitioners in the United States during the summer of 2017. Because the participant’s identity was not tied to the data and because risks to participants for filling out the questionnaire were minimal, the IRB approval fell under the “Exempt” category of review, meaning that an informed consent form was unnecessary (IRB #148–17).

We partnered with the Society of Ecological Restoration (SER) to draw a convenience sample of 200 restoration practitioners randomly selected from their database. A wide variety of types of businesses engage in ecological restoration in the United States, split roughly evenly between the scientific/engineering/design aspects of restoration and the physical construction/earth moving aspects [50, 51]. These firms work with agencies, non-governmental organizations (NGOs), and other stakeholders to execute restoration projects. Given our focus on the business perspective in restoration, the 200 names we drew excluded email addresses that included a.gov or.edu extension. We used a single email solicitation, which included a letter from the SER executive director encouraging participation in our study. The email solicitation stated that participation was voluntary and that responses would remain anonymous. To ensure all respondents based their responses on the domain of ecological restoration, the survey instructions included the Society of Ecological Restoration’s definition of ecological restoration [52].

A total of 115 people clicked on the survey link from the email solicitation. From there, the online survey included three screening questions:

1. Was your ecological restoration experience in the past three years primarily in the US?
  (If no, they did not continue; two respondents did not meet this screen.)
2. Do you run, manage, or work for a company or organization that charges fees and/or earns revenue for providing ecological restoration or ancillary activities (construction, etc.) [as defined previously]?
  (Twelve respondents said they worked with an NGO or government agency that did not earn revenues from providing paid ecological restoration and did not continue the survey.)
3. Do you have direct oversight of, or manage, specific restoration projects?
  (If no, they did not continue; four respondents did not meet this screen.)

Respondents also were told they could skip any question and could quit the survey at any point. The fact that some participants skipped questions resulted in variation in sample sizes among analyses. A total of 97 surveys were completed, for a 48.5% response rate.

### Constructing the survey

We developed questions for the constructs in our study based on studies of innovation in business [4, 53] and then contextualized these to the restoration context. Because they are psychometrically robust in capturing perceptions in survey research [54], we used Likert scales, which allow respondents to specify their level of agreement or disagreement to a series of statements. We pre-tested the questions in individual sessions with three restoration practitioners who sat with the lead researcher as they filled out the survey. Each pre-test respondent spoke out loud about their reactions while completing the questionnaire. We made revisions based on their feedback—for example, a key insight offered was to measure the company’s inclusion of social goals in their restoration work—and the survey was piloted again by the same three practitioners and the research team before being coded into Qualtrics for electronic administration. All questions are detailed in S1 File.

To assess our focal variable of Project-Based Innovation, respondents were prompted to select “The single project within the past three years that you are the most knowledgeable about” and then were asked to agree or disagree with three statements about project-based innovation:

- My company used new techniques on this project.
- My company introduced unproven methods on this project.
- The project objectives were innovative.

If an individual has not used a particular technique before, then that technique would be perceived as novel or innovative to that individual [26, 55]. In conjunction with these three items for Project-Based Innovation, respondents were asked to provide a brief description of the new technique or unproven method in the project.

### Analysis and developing composite measures

Measures are typically assessed for their internal consistency (reliability) as well as dimensionality. For example, multi-item measures (composite indices) are subjected to a factor analysis to assess whether the items load on one factor or if the scale is perhaps comprised of multiple factors (dimensions of the underlying construct). Hence, we used factor analysis to assess the dimensionality of our measures. We also computed Cronbach’s alpha to assess the internal consistency of multi-item measures. For scales of fewer than three items, coefficient alpha is not appropriate [56]; therefore, we computed a Pearson correlation to assess the reliability of any two-item measures. Second, after ensuring that the items loaded on one factor, we created composite indexes for all multi-item scales by summing those items together and dividing by the number of items. Finally, to assess the relationship between each of the sets of variables with the measures of innovation, our analysis relied on a correlational assessment. Although regression analysis would be a stronger test of the relationships between our predictor variables and innovation measures, our relatively low sample size, the option of skipping questions, and correlations between predictor variables (multicollinearity) precluded the use of regression.

## Results

This section first presents a description of the respondents’ traits, followed by the assessment of the measures’ reliability and dimensionality. We then present the results for the Project-Based Innovation, which is followed by the findings of individual, company, and project-level facilitators and inhibitors of innovation. The final set of results are for the relationships between Project-Based Innovation and project outcomes.

### Description of respondents

Table 1 provides a detailed analysis of the respondents’ individual traits and characteristics (Panel A) as well as their companies’ and the projects’ traits and characteristics (Panel B).

**Table 1:** Respondent profile.

**Panel A: Individual characteristics**		
	**Mean/Std. Dev.**	**Median/Range**
**Years in Restoration**	Mean = 19.76 years Std. Dev. = 11.76 (n = 85)	Median = 18.0 years Range = 1–57 years
**Age (n = 70)**	**n**	**%**
20–30 years old	5	7.1
31–40	13	18.6
41–50	20	28.6
51–60	20	28.6
60–70	11	15.7
71+	1	1.4
**Gender (n = 69)**% Male/Female	46/21^a^	66.7%/30.3%
**Area of Expertise** ^b^	**n**	**%** ^c^
Ecological sciences	59	22.4
Plants / botany	57	21.7
Environmental sciences	31	11.8
Forestry and conservation	25	9.5
Water quality	15	5.7
Wildlife biology	13	4.9
Construction	11	4.2
River morphology / design	7	2.7
Civil engineering	6	2.3
Environmental engineering	4	1.5
Other 1^d^	27	10.3
Other 2	7	2.7
Other 3	1	0.4
**Panel B: Company Characteristics**		
	**Mean (Std. Dev.)**	**Median and Range** ^a^
Years working at current company (n = 84)	Mean = 11.06 years	Median = 8.5 years
	Std. Dev. = 9.14 years	Range 1–42
# of FTE at this office location (n = 82)	Mean = 25.99 FTE employees	Median = 12
	Std. Dev. = 35.44	Range 1–150
% of company’s business that is in restoration (n = 68)	Mean = 59.54%	Median = 57%
	Std. Dev. = 34.62	Range 1–100%
# of restoration projects per year (n = 73)	Mean = 14.95	Median = 5
	Std. Dev. = 21.34	Range 1–100
Average project size last three years ($ $) (n = 46)	Mean = $102,521.74	Median = $50,000
	Std. Dev. = $135,698.15	Range 0-$500,000
This project’s size ($ $) (n = 44)	Mean = $516,081.80	Median = $280,000
	Std. Dev. = $819,279	Range = $100-$5,000,000
**Geographic Region** ^b^	**n**	**%** (of 81 responses)
Great Lakes Region	19	23%
Northeastern Seaboard	18	22%
Pacific Northwest	12	15%
Southwest	8	10%
Upper Rocky Mountains	7	9%
California	7	9%
Southeast	6	7%
Great Plains	4	5%
**Project Ecosystem** ^b^	**n**	**% (**of 90 responses)
Wetlands	32	35.6
Forest	20	22.2
Urban	14	15.6
Freshwater Aquatics	14	15.6
Coastal/Estuarine	12	13.3
Grasslands	14	15.6
Arid lands	5	5.6
Marine	2	2.2
Alpine/tundra	2	2.2
Other^c^	13	14.4
**Project Originator/Owner** ^c^	**n**	**%** (of 77 respondents)^d^
Private business	18	23
City/Municipal agency	17	22
State government agency	16	21
Private citizen/landowner	13	17
Federal government agency	12	15.6
Nongovernment Org (NGO)	6	8
Other	12	15.6

^a^ n = 2 (3%) preferred not to answer; n = 21 did not answer/missing

^b^ Area of Expertise exhibited no significant relationship with Project-Based Innovation

^c^ Numbers sum to greater than 100% because respondents could select up to three areas of expertise; 23.3% of respondents identified 1 or 2 areas of expertise; 65.6% of respondents chose 3 areas; and 11.1% chose between 4–8 areas.

^d^ Of the “Others,” the most common areas of expertise were: Architecture, Hydrology, Wetlands, and Horticulture

^a^Outliers on some variables created skewness and hence, were removed from analysis: 3 outliers in # of FTE employees with 200, 350 and 800 FTE; 1 outlier in # of restoration projects per year with 200 projects; 3 outliers in Average project size ($ $) with 700K, 1 mil, and 2 mil

^b^Neither Geographic Region nor Project Ecosystem exhibited significant relationships with Project-Based Innovation

^c^Other included Agricultural, Riparian and Floodplain

^d^ Numbers sum to more than 100% due to multiple responses: 60% of projects had just one “owner,” while 15.5% had two owners; 21% had 2–3 owners, and 4% had 4 owners.

Respondents had, on average, nearly 20 years of experience in restoration. The majority of respondents were between 40 and 60 years of age (57% of respondents), with roughly 67% male and 30% female. Most respondents’ expertise was in the ecological sciences (22.4%) with another 21.7% focused on botany. Roughly 12% had expertise in environmental sciences with another 9.5% focused on forestry and conservation. Respondents had, on average, worked for their company for roughly 11 years. Companies employed roughly 26 FTE employees (median of 12), completed 15 projects per year (median of 5) with a mean project size of just over $100,000 (median of $50,000). (Outliers on these size variables created skewness in our data and hence, we removed seven cases from subsequent analysis: three outliers in size of firm (# of FTE employees with 200, 350 and 800 FTE); one outlier in the number of restoration projects per year (with 200 projects); and three outliers in average project size $ (with 700K, 1 mil, and 2 mil). The companies were located primarily in the northern part of the United States. Wetlands comprised the bulk of the projects (35.6%), with forest ecosystems being the next most common (22.2%). Finally, although the majority of projects were originated by private businesses (23%), collectively, city, state, and federal projects comprised a full 58.6%. Private citizens or landowners accounted for 17% of the projects, and NGOs another 8%. Understanding these sample characteristics helps contextualize our findings.

### Assessment of measures’ internal consistency and dimensionality

Here, we present the psychometric evaluation of our focal construct, Project-Based Innovation. All factor analyses and reliability assessments for other variables appear in the Supporting Information Factor Analyses (S1-S7 Tables in S2 File).

**Fig 2:**
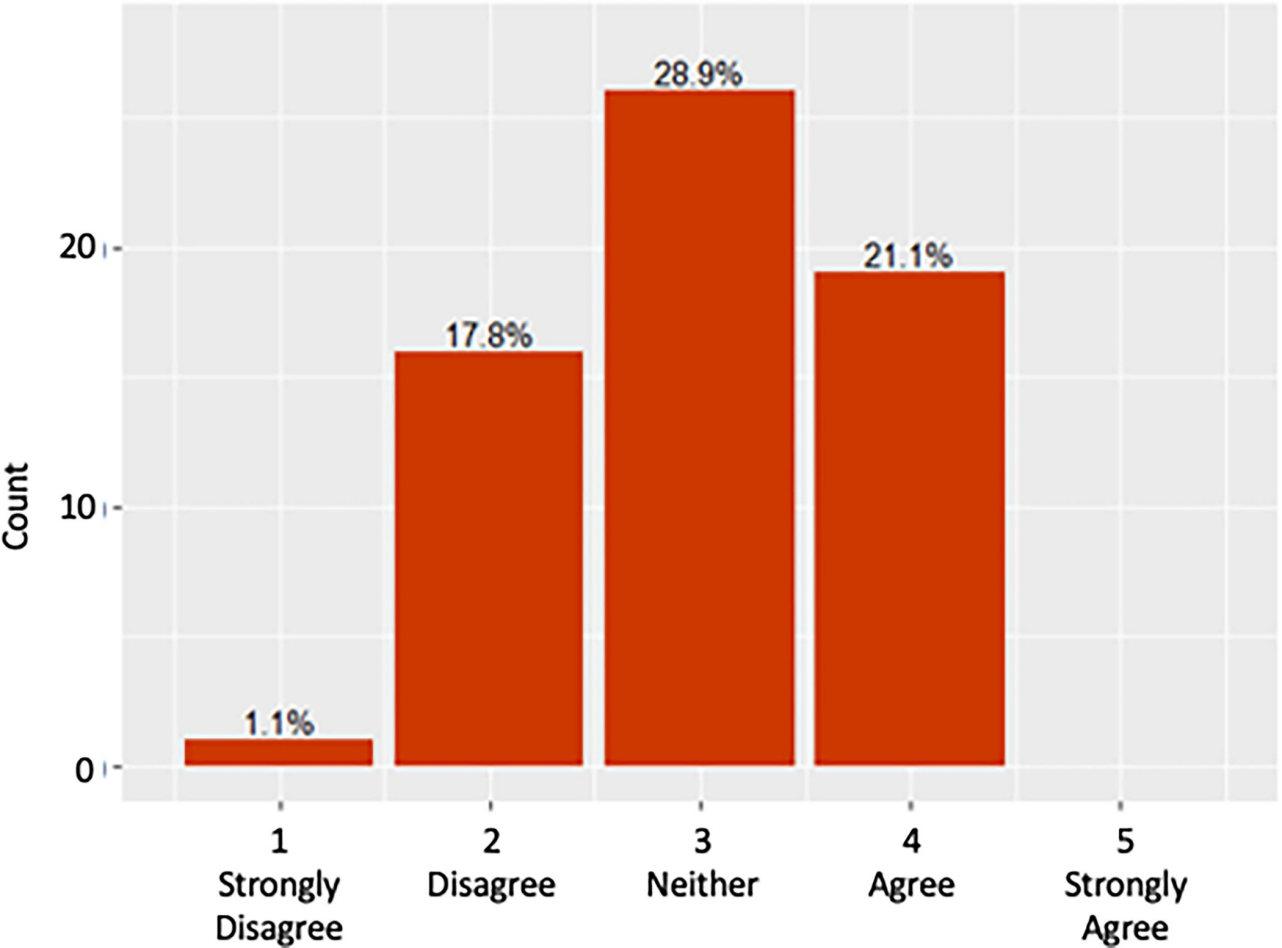
Distribution of responses on project-based innovation.

The three questions used to assemble our key construct *Project-Based Innovation* loaded on a single factor and exhibited a Cronbach’s alpha of 0.77, indicating acceptable reliability. We use this composite index for all subsequent analysis. The mean for Project-Based Innovation (on a five-point scale) was 3.02 (std. dev. = 0.80), with the distribution of responses shown in Fig 2. Notably, no respondents stated “Strongly Agree” in terms of their use of innovations on their projects.

**Fig 3:**
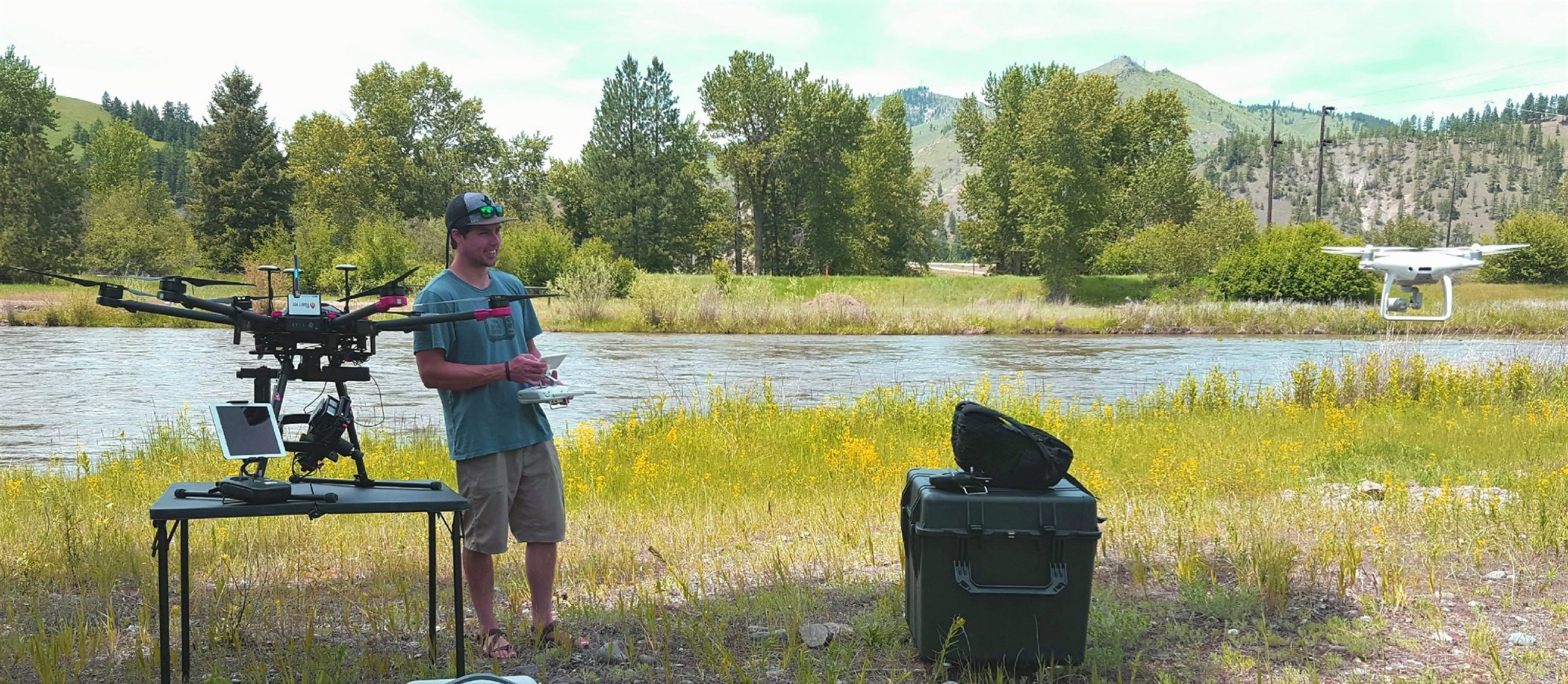
Image of drone being used to measure algal blooms in river.

Respondents were asked: “If your company used new techniques or introduced unproven methods, please provide a brief description of one of them here.” Table 2 offers a sampling of the 28 responses. Numbers do not sum to 28 as some were listed as proprietary/confidential and others were difficult to categorize. Some respondents stated that constraints in cost or design parameters forced them to innovate (n = 4). Other respondents developed innovations in response to dealing with invasive species or urban environments that required novel thinking (n = 3). Still others explained that their innovations applied “fairly standard practice” to a new region or ecosystem for which outcomes were uncertain (n = 6). Other responses identified using technologies such as drones for monitoring, see Fig 3, (n = 1), new software for functional analysis (n = 1), or the use of other new technologies for planning and monitoring restoration (n = 3). One respondent (n = 1) had designed novel equipment for planting. In many cases, responses indicated that the project required trying new methods or approaches for which evidence or guidelines were unavailable.

**Table 2:** Sampling of qualitative descriptions of project-based innovations.

Description:
Used only salvaged material for erosion control and/or wetlands habitat
Creative in designing river channels (fish passages) due to cost
Lack of evidence of how to re-introduce 33 threatened and endangered plant and animal species
Collect seed from adjacent remnant properties in 30-mile radius
Used mulching techniques for invasive removal; new for our area but used in other geographic regions
Drones for monitoring
Innovative large-scale veg propagation to enhance ecological diversity at low cost
Creative stream baffles for erosion control and flood plain access
Lack of evidence on how to introduce native planting in urban environment to maximize diversity of wildlife habitats
Use pre-vegetated mats for trenching in permafrost sites
Changed timing of invasive species control to be more effective
Wetland functional analysis as a design tool where it hadn’t been used before
Re-use veg for erosion;
Recreate vernal pools for endangered species
Roller chipping site to reduce woody shrub coverage; not effective; returned to root raking after a few seasons
New application of old technique for erosion control on riverbank by using shrubs and woody debris to decrease alluvium flow into coral reefs
Phased invasive canopy removal to allow native canopy to adapt to new conditions
Proprietary/confidential

#### Exploring facilitators and inhibitors of Project-Based Innovation

Below, we present the correlations between Project-Based Innovation and the three categories of facilitators and inhibitors of innovation: (a) the individual’s traits, (b) the company characteristics, (c) and the project characteristics. The correlational results report on the 66 participants for which we had complete data. We also present correlations between Project-Based Innovation and project outcomes for the subset of projects that had been completed.

#### Individual-level correlates of innovation

Higher levels of Project-Based Innovation were significantly correlated with greater age (r = 0.30, p < .01) (Table 3). Based on a median split on age (54.3% of respondents were younger than 50, while 45.7% of respondents were 51 or older), older respondents reported higher levels (mean = 3.17 and SD = 0.76) of Project-Based Innovation compared to younger respondents (mean = 2.90 and SD = 0.71). In addition, perceptions of Project-Based Innovation were significantly positively correlated with Years of Experience (r = 0.33, p<0.01). Males were significantly more likely to report that they engaged in Project-Based Innovation than females (r = 0.26, p < .05; mean = 3.18 and SD = 0.74 versus mean = 2.78 and SD = 0.71, respectively). A post-hoc t-test for gender was significant (t = 2.04, p<0.05). Reports of Project-Based Innovation also showed a significant negative correlation with Risk Aversion (r = -0.38, p<0.01). As Risk Aversion increased, Project-Based Innovation decreased.

**Table 3:** Correlational analysis for individual traits and characteristics^a^.

						Frequency of Use of Information Sources					
	Project Innov.	Age	Gender	Yrs. Experience	Risk Averse	Attend Conf/Talks	Talk to colleagues	Journals	Ind. Blogs and trainings	Engage w/ Res. Scientists	Res Scient. Helpful
**Proj. Innov.**	1.00										
**Age**	0.30	1.00									
**Gender (1 = Male)**	0.26	0.41	1.00								
**Yrs. Exper.**	0.33	0.80	0.44	1.00							
**Risk Averse**	-0.38	0.26	-0.32	-0.33	1.00						
**Attend Conf**	0.03	0.09	0.12	0.00	-0.26	1.00					
**Talk to colleagues**	0.01	0.16	0.18	0.13	-0.10	0.28	1.00				
**Journals**	0.12	0.19	0.18	0.44	-0.19	0.10	0.05	1.00			
**Industry info**	-0.35	0.24	-0.05	-0.19	0.19	0.34	0.15	0.17	1.00		
**Engage with Res. Scientists**	0.53	0.17	0.28	0.35	-0.46	0.42	0.13	0.32	0.01	1.00	
**Res. Scientists Helpful**	0.26	0.07	0.05	0.03	-0.31	0.02	0.05	0.02	-0.20	0.38	1.00
**Mean**	3.39	3.32	0.68	21.57	2.48	3.67	5.91	4.71	3.74	2.52	3.79
**Std. Dev.**	0.92	1.20	0.46	12.16	0.75	1.07	0.38	1.43	1.12	0.93	0.61
**Range**	1–5	1–6^b^	0 = Female 1 = Male	1–57	1–5	1–6	1–6	1–6	1–6	1–4	1–5

^a^ Correlations > .30 significant at p < .01; correlations between .24 and .29, significant at p < .05; correlations between .19 and .23, significant at p < .10

^b^ = Categorical variable; See S1 File

Regarding Sources of Information that respondents used, Project-Based Innovation was negatively correlated with the use of industry trainings, blogs, and webinars (r = -0.35, p<0.01); respondents who reported that they relied more heavily on these sources of information reported lower rates of Project-Based Innovation. No other information source factors (attending conferences; talking to colleagues; reading journals) exhibited significant correlations with Project-Based Innovation.

Respondents who engaged with research scientists—and perceived the engagement as helpful—reported higher rates of Project-Based Innovation (r = 0.53, p<0.01 and r = 0.26, p<0.05 respectively). An open-ended prompt to elaborate on the type of engagement with research scientists revealed that practitioners relied on such collaboration to assist in the design of experiments and data collection protocols, collection of specimens, molecular analyses, modeling assistance in estimates for ecological benefits and vegetation growth rates, designing assessment and monitoring plans, and accessing the “state of the science” in their work.

#### Company-level correlates of innovation

Only one company-level variable was correlated with Project-Based Innovation (Table 4): practitioners whose companies had a greater emphasis on social goals reported greater Project-Based Innovation than those whose portfolios had fewer or no projects with social goals (r = 0.25, p<0.05). Non-significant correlates of Project-Based Innovation were company size (FTE), the percent of the company’s business devoted to restoration, and the number of restoration projects per year.

**Table 4:** Correlational analysis for company traits and characteristics^a^.

	Project Innov.	Co Size FTE	% Bus Restoration	# Restor. Projects	Social Goals
**Proj. Innov.**	1.00				
**Co Size FTE**	0.15	1.00			
**% Bus. Restoration**	0.10	-0.17	1.00		
**# Restor. Projects**	0.03	0.18	0.31	1.00	
**Social Goals**	0.25	0.13	0.12	0.09	1.00
**Mean**	3.39	25	58.32	15.08	2.91
**Std. Dev.**	0.92	31.60	32.49	21.64	0.96
**Median**	3.33	14.50	58.32	5.00	3.00
**Range**	1–5	1–135	1–100%	1–100	1–5

^**a**^Correlations > .30 significant at p < .01

Correlations between .24 and .29, significant at p < .05

Correlations between .19 and .23, significant at p < .10

#### Project-specific correlates of innovation

Two project-specific variables were positively correlated with Project-Based Innovation (Table 5). As relative project complexity and project length increased, so did Project-Based Innovation (r = 0.27 and r = 0.26, p<0.05 respectively). More specifically, more complicated projects exhibited a Project-Based Innovation score of 3.13 (std. dev. = 0.71) while easier projects exhibited a Project-Based Innovation score of 2.58 (std. dev. = 0.42). Projects shorter than 10 years exhibited lower levels of Project-Based Innovation (mean = 2.94, SD = 0.64) compared to projects longer than 10 years (mean = 3.15 and SD = 0.70). Five other project variables were marginally correlated with Project-Based Innovation: relative project size (r = 0.20), number of other businesses involved (r = 0.21), collaborative tenor of engagement with other businesses (r = 0.22), perceptions that objectives were helpful (r = 0.20), and uncertainty due to climate change (r = 0.20), all at p<0.10. Uncertainty due to project politics was not significantly related to Project-Based Innovation.

**Table 5:** Correlational analysis for project characteristics^a^.

	Project Innov.	Project Size ($ $)	Project Size (Relative)	Project Complexity	Project Length	# other businesses	Collab. Tenor	Obj. Helpful	Climate-Related Uncertain	Company Experience Uncert.	Project Politics Uncert.
**Project Innov.**	1.00										
**Project Size $ $**	0.18	1.00									
**Project Size (Relative)**	0.20	0.28	1.00								
**Project. Complexity**	0.27	0.27	0.51	1.00							
**Project Length**	0.26	0.12	0.19	0.39	1.00						
**# other businesses**	0.21	0.05	0.22	0.20	0.19	1.00					
**Collaborative Tenor**	0.22	0.07	0.00	0.03	-0.30	-0.20	1.00				
**Helpful Obj.**	0.20	0.15	0.18	0.04	-0.12	-0.02	0.06	1.00			
**Climate-related Uncertainty**	0.20	-0.05	0.22	0.03	0.01	0.01	0.05	-0.13	1.00		
**Company-related Uncertainty**	0.16	0.14	0.08	0.29	0.32	0.05	0.09	-0.13	0.08	1.00	
**Uncertainty due to Project Politics**	0.17	0.15	0.20	0.31	0.16	0.27	0.02	-0.27	0.22	0.17	1.00
**Mean**	3.39	516,081	2.38	2.42	4.28	5.80	3.34	4.23	2.70	1.96	2.11
**Std. Dev.**	0.92	666,360	0.73	0.55	4.20	6.34	0.35	0.72	0.79	0.73	0.94
**Median**	3.33	515,081	3	2.00	3.50	5.00	3.35	4.05	3.00	2.00	2.00
**Range**	1–5	100–5 Mill	1–3	1–3	<1–16 yrs	1–50	1–5	1–5	1–4	1–4	1–4

^a^Correlations > .30 significant at p < .01; between .24 and .29, significant at p < .05

#### Outcomes from project-based innovation

The correlation between Project-Based Innovation and Project Outcomes (completed on-time/on-budget) was not significant (Table 6). Respondents who reported higher levels of Project-Based Innovation reported higher Personal Satisfaction with their work (r = 0.42, p<0.05).

**Table 6:** Correlational analysis for project outcomes^a^.

	Project Innov.	Positive Completion	Personal Satisfaction
**Proj. Innov.**	1.00		
**Positive Completion**	0.05	1.00	
**Personal Satisfaction**	0.42	0.35	1.00
**Mean**	3.36	4.39	4.45
**Std. Dev.**	1.02	0.65	0.91
**Range**	1–5	1–5	1–5

^a^ Due to lower sample size (n = 22 respondents for completed projects), correlations

from .42 - .49, significant at p < .05; correlations between .36 - .41, significant at p < .10

## Discussion

Overall, practitioners rated their Project-Based Innovations near the scale mid-point (e.g., as about average), with no respondents strongly agreeing that their project objectives, techniques and methods were new, unproven, or innovative. Those who reported higher Project-Based Innovations were older, more experienced men who were less risk averse, engaged with research scientists more, perceived such engagement as being helpful, and their companies’ projects included a focus on social goals. Furthermore, more complex and longer projects were correlated with higher levels of Project-Based innovation. Here, we situate these findings relative to related literature, acknowledge our study’s limitations, and offer suggestions for future research.

The finding that older respondents reported higher levels of Project-Based Innovation is inconsistent with theory for the adoption of innovation, which posits that adopters of new innovations are generally younger [26]; see also Prokopy, et al’s review [31] which also found that older farmers are less likely to adopt agricultural conservation practices. Perhaps in the field of restoration, age engenders greater confidence, giving older practitioners the necessary perspective to experiment with new techniques and novel methods. This possible explanation is supported by our finding that more experienced practitioners reported higher levels of Project-Based Innovation. (Recall from Table 3 that age and experience were highly correlated (r = 0.80).

On the other hand, consistent with innovation theory [26] and our hypothesis, males reported higher levels of Project-Based Innovation than females. Given that in our study, women skewed younger than men, and because gender is significantly correlated with experience in our study (with men tending to have more experience than women), it is difficult to get a clear picture of the relationship between Project-Based Innovation and gender relative to age and experience. The interplay between these variables would be an interesting area of research, particularly in light of Prokopy, et al. [31], who found women were more likely than men to adopt novel agricultural conservation practices.

The strong negative relationship between Risk Aversion and Project-Based Innovations is consistent with our hypothesis and prior research showing that people who are risk averse are less likely to innovate [32]. Consistent with Mohr and Metcalf’s [24] recommendations based on qualitative findings, our study reinforces the value of encouraging restoration practitioners to be aware of their own risk aversion and to balance it with a willingness to try new things.

The negative relationship between the frequency of usage of industry trainings, blogs, and webinars as sources of information and Project-Based Innovation is intriguing. Typically, adopters of new innovations tend to rely more extensively on industry sources of information [26]. In contrast, our research shows that in the field of ecological restoration, practitioners who relied on these sources of industry-based information were less likely to engage in Project-Based Innovation. Perhaps industry-based information reinforces existing industry practices rather than encouraging novel techniques. Teasing out this relationship would be a fruitful area for additional inquiry.

In contrast to the negative relationships between use of industry-related information and Project-Based Innovation, practitioners who engaged more extensively with research scientists, and who found such engagements helpful and valuable, reported greater Project-Based Innovation. Although scientists are sometimes criticized for being out of touch with “on-the-ground” realities of practitioner needs [24, 57], our findings suggest such collaboration can offer an important source of new insights and methods. Exploring venues and opportunities to facilitate such engagement would be valuable to sparking innovation in ecological restoration.

Our finding that a company’s inclusion of social goals in their work is positively related to Project-Based Innovation suggests that broadening the scope of restoration projects beyond ecological domains can stimulate novel techniques and thinking. The underlying principles of ecological restoration state the importance of stakeholder engagement and social goals [58]. The specific mechanisms by which social goals engender innovation warrants further investigation. Perhaps social goals provide a new lens through which restoration practices are viewed, leading to questioning standard practice and lending new insights. Or perhaps the inclusion of social goals provides greater engagement with other stakeholders, in turn generating opportunities for co-discovery and co-innovation of on-the-ground techniques [59]. Or possibly restoration ecologists who are open to social goals may also be open to innovation.

Prior studies of innovation in business suggest that complexity can inhibit innovation [48] and longer projects may have greater unknowns. However, our study found positive relationships between both project complexity and project length with Project-Based Innovation. Perhaps longer timelines allow, or even facilitate, more experimental thinking. Similarly, perhaps more complex projects spark more out-of-the-box thinking. Looking forward, awareness of project complexity and longer time frames might stimulate innovation and creative thinking to emerge from restoration practitioners.

Finally, our finding that an individual’s personal satisfaction with their work on the project is positively related to Project-Based Innovation suggests that using novel techniques and new methods offers a sense of gratification and fulfillment. However, given our cross-sectional data, it is also possible that someone who really enjoys their work might be more likely to innovate. This finding is particularly noteworthy, given that Project-Based Innovation was unrelated to whether the project was completed on time or on budget. In other words, even if a project’s outcomes were not positive, individuals experienced greater sense of satisfaction with more innovative projects. Practitioners should consider how to best allow for innovation where staff feel safe to experiment and are rewarded for innovative thinking. The finding that Project-Based Innovation was not correlated with project outcomes is perhaps not surprising, given that some innovations are likely to contribute to improved outcomes while other innovations are likely to not work out as expected.

The large number of non-significant correlations is consistent with Prokopy et al. [31], whose review of 93 studies revealed that few independent variables have a consistent relationship with farmers’ adoption of conservation practices. Likewise, the non-significant relationships in our sample could be a function of the high variance in our sample, for example, the wide variety of backgrounds, training and the types of ecosystems in which the restoration was conducted. We expected that by relying on individual respondents’ perceptions of innovation (cf. [60]), our measures would be able to handle such nuances and subtleties. However, various types of innovations—whether new products or technologies (i.e., new type of sensor), new procedures or methods (e.g., genomics or algorithms), or even new approaches (public/private partnerships)—might exhibit different correlates. Additionally, different types of restoration practitioners (e.g., wildlife managers vs. civil engineers) likely experience different facilitators of and barriers to innovation. Similarly, restoration on government contracts might exhibit different triggers of and propensities for innovation than restoration of private lands. Whether our U.S.-based findings might differ from other geographic regions is also an open question. Understanding these and other contextual nuances offers the potential for additional insights into facilitators and barriers of innovation in ecological restoration.

We relied on a well-established approach to measuring perceptions in social science research: Likert scales. It would be interesting to consider other ways to assess innovation in this context as well, for example through measures of expert assessment of the novelty of particular designs and practices. Moreover, we encourage future research on specific restoration innovations other than the project-based innovations our measures focused on. For example, social innovations, such as public and private partnerships or processes for engagement and co-innovation, may be as important as ecological innovations in coping with challenges in ecological restoration.

Another approach to studying the use of innovations in ecological restoration would be to consider specific messaging or communications strategies that might be used proactively to stimulate adoption of innovation. For example, Prokopy et al. [31] suggested the importance of tailoring different messages to different types of farmers to stimulate adoption of conservation measures. Although our study examined sources of information that practitioners rely on, we did not examine the content or strategies those sources of information conveyed. Specific communication strategies, such as those used to facilitate comparisons to others’ behaviors, can be used to “nudge” people along the path to adoption [61, 62], a potentially useful approach to upscaling innovation in ecological restoration. Related to communication strategies, social networks are an important aspect of influence and information (e.g., [29]). Given that different types of network relationships exert different levels of influence on individuals’ behavior, it would be helpful to explore the impact of individuals’ social connections on their use of innovation in ecological restoration.

An issue related to individual practitioner propensity to use innovation is the diffusion of innovation across a discipline, sometimes referred to as “scale up” or uptake. Based primarily on adoption and diffusion of innovation [26], research in the related discipline of conservation has used both case studies [30] as well as quantitative modeling [63] to explore how innovations diffuse throughout a particular discipline (see also [64, 65]). Similar methods might be valuable in the domain of ecological restoration to assess the adoption of particular innovations more broadly

Finally, the relationship between “best practices” in a discipline and innovation offers the potential for valuable insights [66]. Best practices are often codified in industry standards regarding how to perform a particular task or function. The role of such standards, particularly in young fields, may lead to early reification of practices and techniques that might stymie novel thinking [67]. Finding the sweet spot between promoting standards that improve the practice of ecological restoration [58, 68], while at the same time encouraging innovation, would offer fruitful results in ecological restoration.

Certainly, innovations may not always be beneficial. Anticipated or desired outcomes may not occur; for example, one respondent in our study (see Table 2) noted that the innovation used to reduce woody shrub coverage had not been effective. Uncertainty around outcomes is a hallmark of innovation [69, 70]. Also of concern is the possibility of negative outcomes—including those that are either unintended and/or unanticipated. For example, one innovation that offers much promise, and also concern, in restoration is genomics—a suite of technologies that can analyze very large collections of genes within an organism or organisms (e.g., the use of high-throughput DNA sequencing technologies) [17]. Some worry about possible unintended consequences of gene editing and express worries about the “cart being before the horse” with respect to testing out new technologies in a safe fashion [18]. The debates around innovations that offer much promise yet are risky and unproven are both philosophical [71, 72] and practical. On the practical side, risk assessment frameworks for emerging technology exist and these could be adapted for the ecological restoration context (see [17], for example). We encourage greater conversation within the field of ecological restoration around responsible innovation, much like that which is happening in other disciplines [73, 74].

## Conclusion

In summary, our findings uncovered several factors that may facilitate or inhibit project-based innovation in ecological restoration. Older, more experienced practitioners are more likely to report the use of innovations in their restoration projects, while those with greater risk aversion are less likely to report innovative solutions in their work. In addition, individuals who engage with research scientists, and who find those engagements helpful, report greater use of innovative solutions, while those who access industry information report less innovative solutions. Those who work for companies that include social goals report the use of innovations in their restoration projects, as do those who work on more complex, longer projects. Finally, Project-Based innovation is related to satisfaction with the work, indicating either that innovation results in greater satisfaction or that satisfied individuals are more open to innovation. Regardless, innovation appears to be a meaningful aspect of an individual employee’s work. Based on these findings, we offer the following recommendations to enhance innovation in ecological restoration:

- To stimulate innovative practices in early-career practitioners, collaboration between older and younger practitioners may be beneficial.
- To increase rates of innovation, practitioners should be aware of their own risk aversion and attempt to balance it with a willingness to try new things; their companies may also consider ways to decrease the negative consequences of risk for their employees.
- To generate new techniques and practices, engagement with research scientists and inclusion of social goals in ecological restoration can offer favorable opportunities.
- Longer and more complex projects may increase opportunities for innovation.

Our hope is that these insights encourage practitioners to understand their own aptitude for innovation and to explore new approaches to ecological restoration to meet the challenges of the current Decade of Ecological Restoration [22, 75, 76]—and to do so responsibly.

## Supporting information

S1 Data (XLSX)

S1 File Survey questions. (DOCX)

S2 File (DOCX)
