# Supplementary material for: Age, experience, social goals, and engagement with research scientists may promote innovation in ecological restoration
Source: PLoS One. 2023 Apr 25;18(4):e0274153. doi: 10.1371/journal.pone.0274153 (PMC10128931; doi:10.1371/journal.pone.0274153)
Supplement: S1 File — (DOCX) [file pone.0274153.s002.docx]

**Introduction: The Business Perspective on Restoration**

**This research is designed to study businesses that conduct ecological restoration.** In particular, we are interested in your perspective on how your company navigates the complexities of ecological restoration.

**Ecological restoration** – “the process of assisting in the recovery of ecosystems that have been degraded, damaged, or destroyed” (SER 2004) – has become **an important business sector**, with an annual investment of over three trillion dollars worldwide (Cunningham 2002).

Much of **the practice of restoration is conducted by businesses**—consulting firms, engineering firms, nurseries, construction companies and earth movers, etc. Businesses engaged in ecological restoration **interact with a variety of stakeholders**—government agencies, nongovernmental organizations, citizen collaboratives, landowners, recreationists— to complete projects on time and on budget, and to achieve ecological, social, and business objectives.

We would be most grateful if you would be willing to answer our questionnaire. We will share a white paper/summary of the results with you; *the last page of the questionnaire will ask for your email address and this final entry will be kept separate from your responses to maintain the anonymity of your responses.*

Your responses will remain anonymous—only aggregate statistics will be reported from the dataset.

**Ecological Restoration Defined for this Study:**

Ecological restoration, as defined by the Society for Ecological Restoration, refers to the intentional alteration of a site to emulate the structure, functioning, diversity, and dynamics of the specified ecosystem.

Other activities that can be restorative to ecosystems include **remediation, rehabilitation, and reclamation**. These activities share the goal of repairing damaged or degraded ecosystem functions, in order to recreate autonomous or self-sustaining ecosystems, characterized by biotic change or succession in plant and animal communities, energy flow and nutrient cycling, and the ability to repair themselves following natural or moderate human perturbations.

This survey will use the phrase “ecological restoration” to refer to this broad domain.

**Screening Questions and individual position/background:**

1. Has your ecological restoration experience in the past three years been primarily in the U.S.?

Yes (please proceed to Q3).

No

Don’t know/unsure

1. Given the unique context in which ecological restoration occurs, our survey is currently based on ecological restoration work in the United States. If you are working outside the U.S. on ecological restoration, would you like to participate in our survey at a later date:

Yes

No

(end survey with a thank you/option to sign up for copy of results if desired)

1. Do you run, manage, or work for a company or organization that charges fees and/or earns revenues for providing ecological restoration or ancillary activities (construction, etc.) as defined above?

Yes (please proceed to Q5)

No: The company I work for, run, or manage does not charge fees or earn revenues for providing ecological restoration services. (For example, you might work with an NGO or a government agency, or you might be a volunteer.) (proceed to Q4)

1. Please specify how you are involved in the field of ecological restoration: (open ended)

(end survey with a thank you/sign up for copy of results if desired.)

1. What is your job title/position within your company or organization?
2. Do you have direct oversight of, or manage, specific restoration projects?

Yes

No

Not Sure

1. What is your area of expertise (background, training)? Check up to three that are most directly relevant to your expertise, background, and training.

Civil engineering

Construction

Environmental engineering

Ecological sciences

Forestry and conservation

Environmental sciences

Wildlife biology

Plants/Botany

Aquatic

River morphology/design

Other (please describe): ______________________

Other (please describe): ________________

Other (please describe): ___________________

1. In what year did you work on your first restoration project? ___________
2. How many years of total experience do you have working in ecological restoration? _______
3. Where is your company headquartered? (city/state/ zip code)
4. How many years have you worked for your current employer?
5. **With respect to the specific office/business location where you work**: approximately how many employees work at that location on an FTE (full-time equivalent) basis?

Approximate Number: __________

I don’t know

***Section 1:*** For this section, please consider the current company location at which you work, over the **past three year time period.** If you don’t know the answer to any of the following questions, please enter “Don’t know/unsure.”

1. Over the past three years, approximately what percentage of your company’s total work portfolio is focused on ecological restoration or similar activities:
   ________ %

Don’t know / unsure

1. What is the average number of restoration projects per year that you personally have worked on?

______________ (specify number)

Don’t know/unsure

1. Over the past three years, what is the average size of ecological restoration project that your current company location has worked on? (this is your company’s specific piece of the total project).

$ ___________

Don’t know/unsure

**There are no right or wrong answers, as companies and individuals will vary in the degree to which the following statements are applicable to your company’s situation.**

**Although some of these questions may seem repetitive, multiple measures are required to reliably assess perceptions.^[[1]](#footnote-1)^**

Please answer how strongly you agree or disagree with the statements on a five-point scale, with

1 = Strongly Disagree, 2= Disagree, 3 = Neither Disagree nor Agree, 4 = Agree, 5 = Strongly Agree (“don’t know/can’t answer/unsure/not relevant for my position” option).

1. General Perceptions on risk

- I take minimal risks to avoid potential negative outcomes.
- I try to avoid risk at all costs.
- It is not worth it to take substantial risks just for the hope of achieving a positive outcome.

1. Some ecological restoration projects have goals oriented towards society as a whole, and they might include things like public use and recreation, economic livelihoods from the land, and so on. These are sometimes referred to as “**social goals**.”

In the past three years, to what extent have the projects you worked on had social goals (e.g., public use and recreation) as part of the purpose of the ecological restoration?

1. = Not at all/ 2= A minimal extent/ 3 = A moderate extent / 4 = A great extent
2. There are a variety of ways you might stay up to date in your field. For the following sources of information, please indicate the frequency with which you use them.

(1= Never; 2 = Less frequently than once every two years; 3 = Once every two years

4 = Once a year; 5 = Twice a year; 6 = Once a quarter or more frequently)

Professional conferences in my field

Academic or university sponsored talks/presentations

Talking to other colleagues/professionals in the discipline:

Online webinars/trainings

Practitioner journals/magazines

Academic journals like *Restoration Ecology*

Industry blogs/online resources

Trainings by private companies such as the Wetlands Institute or the Rosgen Center.

Other sources of information (please specify and then rate frequency)

Other:

Other:

1. In the past three years, to what extent have you engaged with research scientists (i.e., non-practitioners) in the course of doing your ecological restoration work?
2. = Not at all/ 2= A minimal extent/ 3 = A moderate extent / 4 = A great extent

For those who circled 3 or 4: Please provide an example of how you have engaged with research scientists in doing your ecological restoration work: ______________________________

1. In general, how would you characterize your engagement with these research scientists? Please answer how strongly you agree or disagree with these characteristics.

(5 point scale/Strongly Disagree/Strongly Agree with don’t know/unsure option)

Helpful

Cutting edge

Realistic

Spot on

Valuable

**Section 2:** For the following questions, please select **the single project within the last three years** that you are most knowledgeable about.

What was the project’s name/how did you refer to the project: ___________________

1. Please tell us some of the details of that particular project. For that specific project:
2. When did it start? (month/year)

I don’t know.

1. When did it end/when is it projected to be done? (month/year)
   I don’t know.
2. What was your company’s portion of its planned budget in $$? _________________

I don’t know.

1. Relative to other projects you have worked on in the past, is this project:

Smaller Typical Larger

Easier Typical More Complicated

1. In what type of ecosystem was the project situated? Please select up to two ecosystems that are most directly relevant to this project.

forest

wetlands

freshwater aquatic

marine

coastal / estuarine

arid lands

grassland

alpine/tundra

urban

other (please specify):

other (please specify)

1. Regardless of oversight or who you interact with, who was the originator / owner of this project? (check all that apply)

Federal government agencies

State government agencies

City/Municipal agencies

Nongovernmental organizations (NGOs)

Private businesses

Private citizens/landowner/individual

Other (please describe):

Don’t know/unsure

1. For this particular project, what is your best estimate of how many other businesses in total were involved, including primes or subs? For example, these businesses might include nurseries/greenhouses, heavy equipment or construction companies, engineering firms, and so on.

_________________#

Don’t know/not sure

1. What was the tenor of engagement/collaboration **between the various businesses** engaged on this particular project? Please answer how strongly you agree or disagree with the following statements.

1 = Strongly Disagree, 2= Disagree, 3= Neither Disagree nor Agree, 4 = Agree, 5 = Strongly Agree (don’t know/unsure option).

- In general, the various businesses worked well together.
- The various business seemed to step on each other’s toes.
- Communication between the various parties doing the work was effective.
- The parties effectively worked through differences

1. Were restoration goals articulated for this project?

Yes

No (go to Q7)

I don’t know (go to Q7)

How would you characterize the project objectives? Please answer how strongly you agree or disagree with the following statements.

(5 point scale/Strongly Disagree/Strongly Agree with don’t know/unsure option)

- They allowed us to do high quality work.
- They were realistic.

1. Uncertainty is a natural element of most restoration projects. Please rate the following dimensions in terms of the level of uncertainty they introduced on this specific project.

(4 point scale: 1= introduced no degree of uncertainty 2= introduced a low degree of uncertainty; 3 = introduced a moderate amount of uncertainty 4 = introduced a high degree of uncertainty)

- The vagaries of nature (floods, weather, drought, fire)
- Climate change induced uncertainties
- Experience of my company with this type of restoration project
- Restoration methods/protocols for this project domain
- Disagreements between/ among various stakeholders/businesses/organizations/citizen collaboratives, etc.
- Politics external to the project

1. How would you characterize the company’s work on this project? Please answer how strongly you agree or disagree with the following statements. My company:

- Used new techniques
- Introduced unproven methods
- Had innovative project objectives

1. If your company used new techniques or introduced unproven methods, please provide a brief description of one of them here:
2. Has your company’s work on this project been completed?

Yes (If yes, complete next two questions)

No (go to respondent demographics)

Don’t know/unsure (same)

11. **For projects that are completed,** please answer how strongly you agree or disagree with the statements. I would say my company’s overall work on this project:
(5 point scale/Strongly Disagree/Strongly Agree with don’t know/unsure option)

- was completed on schedule
- was completed within the budget
- accomplished the project design goals
- met major milestones as planned

12. **How satisfied are you, personally, with your work on this project?**

1 = Extremely dissatisfied to 5 = Extremely satisfied

**Section 3: End of survey respondent background/questions:**

1. How old are you:

20-30

31-40

41-50

51-60

61-70

71+

1. What is your gender:

Male

Female

Prefer not to answer

1. Is there anything else you would like us to know about the business of restoration? For example, what question did you expect to be asked, but were not? What do you want us to know that we did not ask?

Your time is incredibly valuable. Thank you very much for providing your responses to this survey.

If you would like a copy of the results from this project, please enter your email address here: (this will be kept separate from the rest of your questionnaire to maintain your anonymity)

_________________________________

Thank you. If you have any additional questions or comments, please feel free to email me at [Jakki.mohr@business.umt.edu](mailto:Jakki.mohr@business.umt.edu)

Warm regards,

Jakki J. Mohr, Ph.D.

Regents Professor of Marketing
Poe Family Distinguished Faculty Fellow

Fellow, Montana Institute on the Environment

School of Business Administration

University of Montana

Missoula, MT 59812

1. The original questionnaire included several other variables based on Mohr & Metcalf (2018); however, these variables exhibited poor psychometric properties and were not included in our analysis. [↑](#footnote-ref-1)
